# Barnidipine or Lercanidipine on Echocardiographic Parameters in Hypertensive, Type 2 Diabetics with Left Ventricular Hypertrophy: A Randomized Clinical Trial

**DOI:** 10.1038/srep12603

**Published:** 2015-08-05

**Authors:** Giuseppe Derosa, Amedeo Mugellini, Fabrizio Querci, Ivano Franzetti, Rosa Maria Pesce, Angela D’Angelo, Pamela Maffioli

**Affiliations:** 1 Department of Internal Medicine and Therapeutics, University of Pavia, Fondazione IRCCS Policlinico San Matteo, Pavia, Italy; 2 Center for the Study of Endocrine-Metabolic Pathophysiology and Clinical Research, University of Pavia, Pavia, Italy; 3 Laboratory of Molecular Medicine, University of Pavia, Pavia, Italy; 4 Ospedale Pesenti Fenaroli, Alzano Lombardo, Bergamo, Italy; 5 Metabolic Unit, S. Antonio Abate Hospital, Gallarate, Varese, Italy; 6 PhD School in Experimental Medicine, University of Pavia, Pavia, Italy

## Abstract

The aim of this study was to evaluate the effects of lercanidipine or barnidipine on echocardiographic parameters, in hypertensive, type 2 diabetics with left ventricular hypertrophy. One hundred and forty-four patients were randomized to lercanidipine, 20 mg/day, or barnidipine, 20 mg/day, in addition to losartan, 100 mg/day, for 6 months. We evaluated: blood pressure, fasting plasma glucose (FPG), glycated hemoglobin (HbA_1c_), lipid profile, creatinine, estimated glomerular filtration rate (eGFR), sodium, potassium, and acid uric. Echocardiography was performed at baseline and after 6 months. Both lercanidipine and barnidipine decreased blood pressure. Left ventricular mass index was reduced to a greater extent with barnidipine + losartan. Interventricular septal thickness in diastole was reduced by barnidipine + losartan. Posterior wall thickness in diastole was decreased by both treatments, even if barnidipine + losartan were more effective. Ratio of peak early diastolic filling velocity to peak filling velocity at atrial contraction was increased by barnidipine + losartan, but not by lercanidipine + losartan. Finally, isovolumetric relaxation and time and left atrial volume index were reduced by barnidipine + losartan, while lercanidipine + losartan did not affect them. In conclusion, barnidipine + losartan provided a greater improvement of echocardiographic parameters compared to lercanidipine + losartan.

Left ventricular hypertrophy (LVH), a marker of cardiac end-organ damage, is frequently found in hypertensive patients and has been recognized to predict cardiovascular complications more strongly than other risk factor except for advancing age^1^,^2^. Diabetes mellitus has been demonstrated to be an independent stimulus for LVH, that may contribute to cardiovascular events in diabetic individuals^3^,^4^. When hypertension is associated with diabetes, as it frequently happens, the development of LVH is further accelerated and the risk of cardiovascular complications is greatly enhanced^5^. A large proportion of patients with type 2 diabetes and no known cardiovascular disease have left ventricular hypertrophy^6^; moreover, patients with type 2 diabetes mellitus are at 2–5 folds higher risk for developing heart failure^7^. There is evidence that regression of LVH by pharmacological intervention is associated with an improvement in prognosis, independent of how much the blood pressure (BP) is lowered^8^,^9^; however, the various anti-hypertensive agents may differ in their ability to regress LVH. A review recently published^10^ asserted that the best treatment of LVH is its early identification and rapid implementation of an adequate treatment with greater prevalence of LVH. Angiotensin II receptor blockers (ARBs) and angiotensin-converting-enzyme inhibitors (ACE-inhibitors) should be the first line therapy, because they proved to be the most effective in reducing LVH in type 2 diabetic patients. Where ACE-inhibitors or ARBs are contraindicated or not tolerated, or when they are not enough to reach an adequate blood pressure control, another anti-hypertensive agent should be added, and calcium channel blockers (CCBs) could be an option as second line therapy^10^. At this regard, barnidipine hydrochloride is a 1,4-dihydropyridine CCB with long-lasting vasodilatory effect^11^,^12^. Its anti-hypertensive action is related to the reduction of peripheral vascular resistance^11^,^12^. Lercanidipine is a third-generation dihydropyridine CCBs that causes systemic vasodilation by blocking the influx of calcium ions through L-type calcium channels in cell membranes. It is a highly lipophilic drug that accumulates in the lipid bilayer of cell membranes in the arterial wall compartment, which causes slow drug redistribution from this tissue and promotes a slow onset of action^13^.

Calcium channel blockers did not have the same effect on LVH, for this reason the aim of this study was to evaluate the effects of lercanidipine compared to barnidipine, in addition to losartan, on some echocardiographic parameters, in hypertensive, type 2 diabetic patients, with LVH.

## Material and Methods

### Study design

This multicenter, randomized, double-blind, controlled study was conducted at the Department of Internal Medicine and Therapeutics, University of Pavia, Fondazione IRCCS Policlinico S. Matteo, PAVIA, Italy (coordinating site); Ospedale Pesenti Fenaroli, Alzano Lombardo, BERGAMO, Italy; Metabolic Unit, S. Antonio Abate Hospital, Gallarate, VARESE, Italy.

The study protocol was conducted in accordance with the Declaration of Helsinki and its amendments, and the Good Clinical Practice Guidelines. It was approved by the each Ethical Committees and all patients provided written informed consent prior to entering the study. TRIAL REGISTRATION: ClinicalTrials.gov NCT02064218.

## Patients

We enrolled 144 mild to moderate hypertensive, type 2 diabetic patients, with LVH, not well controlled by losartan, 100 mg/die, with low density lipoprotein cholesterol (LDL-C) <160 mg/dl), overweight outpatients, aged ≥18 years, of either sex (Table 1).

Patients were evaluated for eligibility according to the following inclusion criteria:

- systolic blood pressure (SBP) ≥ 140 mmHg < 180 mmHg and/or diastolic blood pressure (DBP) ≥ 90 mmHg < 105 mmHg
- well controlled type 2 diabetes mellitus (HbA_1c _≤ 7.5%)
- LVH defined as a LVM index (LVMI) >134 g/m^2^ in men and >110 g/m^2^ in women.

The exclusion criteria were secondary hypertension, severe hypertension (SBP ≥ 180 mmHg or DBP ≥ 105 mmHg), hypertrophic cardiomyopathies due to etiologies other than hypertension, history of heart failure or a LV ejection fraction (LVEF) ≤ 50%, history of angina, stroke, transient ischemic cerebral attack, coronary artery bypass surgery or myocardial infarction any time prior to visit 1, concurrent known symptomatic arrhythmia, liver dysfunction (AST or ALT values exceeding 2-fold the upper limit), creatinine >1.5 mg/dl, known hypersensitivity to the study drugs. Pregnant women as well as women of childbearing potential were excluded.

Suitable subjects, identified from review of case notes and/or computerized clinic registers were contacted personally or by telephone.

### Treatments

The patients fulfilling the inclusion and exclusion criteria, were randomized to lercanidipine 20 mg/day, or barnidipine, 20 mg/day, in addition to losartan 100 mg/day for 6 months. To ensure the blind status of the study, lercanidipine, and barnidipine were supplied as identical, white capsules in coded bottles. Randomization was done using envelopes with codes prepared by a statistician who was the only one to have a copy of the code. The code was broken after database lock, but could have been broken in cases of an emergency. Medication compliance was assessed by counting the number of pills returned at the specified visit. At baseline, participants received two bottles containing a supply of the study medication for at least 100 days. Patients were instructed to take their first dose of new medication on the day after they were given the study medication. All unused medication was retrieved for inventory.

### Diet and Exercise

Patients were already following a controlled-energy diet; for a description of the diet followed by patients, see our previous work^14^.

### Assessments

Before starting the study, all patients underwent an initial screening assessment that included a medical history, physical examination, vital signs, and a 12-lead electrocardiogram. We evaluated at baseline, and after 6 months these parameters: body weight and body mass index (BMI), SBP and DBP, heart rate (HR), fasting plasma glucose (FPG), glycated haemoglobin (HbA_1c_), serum creatinine, estimated glomerular filtration rate (eGFR), sodium, potassium, total cholesterol (TC), triglycerides (Tg), high density lipoprotein cholesterol (HDL-C), LDL-C, acid uric. Clinic blood pressure was measured every month, while conventional echocardiography was performed at the baseline and at the end of 6 months of treatment.

For a description of how various parameters were assessed, see our previous work^15^.

Glycated hemoglobin level was measured by a high performance liquid chromatography method (DIAMAT, Bio-Rad, USA; normal values 4.2–6.2%), with intra- and interassay coefficients of variation (CsV) of <2%^16^.

### Echocardiography

Transthoracic echocardiographic assessment was performed by one physician blinded to study treatment, certified in adult echocardiography by the National Board of Echocardiography, with at least five years of experiences in performing echocardiography. Patient was maintained in the left lateral decubitus position, using an ultrasound machine with a 2- to 4-MHz transducer. Echocardiograms were obtained in a standard manner using standard parasternal, short axis, and apical views. Doppler recording were obtained using a phase-array echo-Doppler system. Measurements were made with a computerized review station equipped with digitizing tablet and monitor screen overlay for calibration and measurement performance. Standard M-mode and 2-dimensional measurements were taken according to the guidelines of the American Society of Echocardiography^17^. Left ventricular ejection fraction was measured by the quantitative 2-dimensional biplane modified Simpson method from a 4- and 2-chamber view. The LVM was calculated according to the previous study of Devereux *et al.*^18^). The LVM was divided by body surface area to calculate the LVMI. Pulsed Doppler recordings were made from the standard apical four-chamber view. Mitral inflow velocity was recorded with the sample volume at the mitral annulus level; the average of the three or more cardiac cycles was taken. The following measurements were made: peak velocity of early ventricular filling (E), peak velocity of late ventricular filling (A) and their ratio (E/A), and left atrial volume index (LAVI).

### Statistical Analysis

Data are expressed as mean ± standard deviation (SD). The statistical analysis of the data was performed by the statistical analysis software (SAS) system, version 6.12 (SAS Institute, Inc., Cary, NC, USA). The differences between the two groups in baseline characteristics were analyzed by the two-tailed Student’s *t*-test. Comparisons within and between groups were assessed by a two-way ANOVA for repeated measurements. Differences between baseline and after 6-months’ treatment in each group in blood pressure and echocardiographic determinations were analyzed with the Wilcoxon signed rank test. Comparisons of changes in blood pressure and echocardiographic determinations between the two groups were performed with the Mann-Whitney U-test^19^. Findings of p < 0.05 were considered significant. Considering as clinically significant a difference of at least 10% compared with the baseline and an alpha error of 0.05, the actual sample size was adequate to obtain a power higher than 0.80 for all measured variables.

## Results

### Study sample

One hundred and forty-four patients were enrolled; 73 were randomized to lercanidipine and 71 to barnidipine. One hundred and thirty-nine patients completed the study. Five patients did not complete the study and the reasons for prematurely withdrawal included: lost to follow-up (3 patients), and withdrawal of informed consent (2 patients). No patients interrupted the study due to adverse events. As regards concomitant medications, 83% of patients treated with barnidipine and 85% of patients treated with lercanidipine were taking hypo-cholesterolemic agents, without significantly difference between the two groups, and their dosage was maintained stable during the study.

### Blood pressure

**Figure 1:**
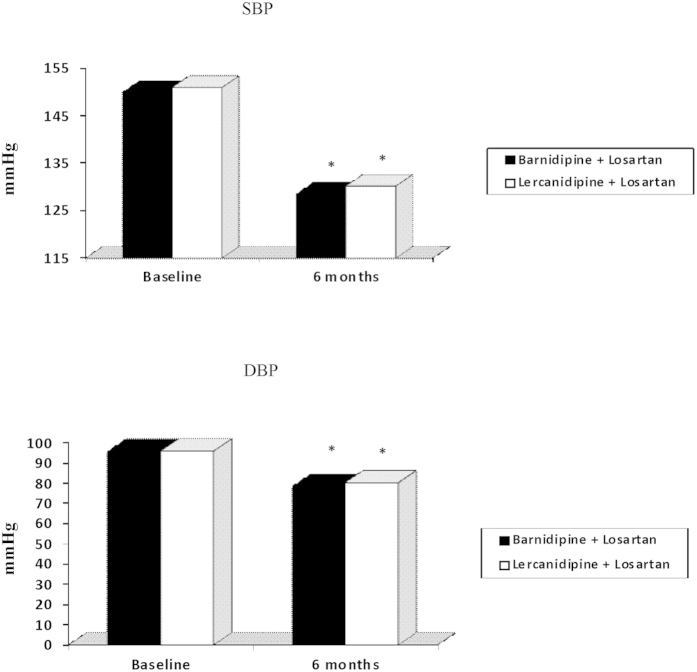
*p < 0.001 vs baseline. SBP: systolic blood pressure; DPB: diastolic blood pressure.

Both lercanidipine and barnidipine induced a similar, significant SBP and DBP reduction (p < 0.001 vs baseline for both), with no statistically significant differences between the two groups (Fig. 1).

### Metabolic parameters

Metabolic parameters were not affected by neither of treatments with the exception of LDL-cholesterol that was reduced by barnidipine + losartan (p < 0.05 vs baseline and vs lercanidipine + losartan), and acid uric that was reduced in both groups (p < 0.05 compared to baseline for both) (Table 2).

### Echocardiographic parameters

Left ventricular mass index was reduced by both treatments, but to a greater extent with barnidipine + losartan (p < 0.05 vs lercanidipine + losartan). Interventricular septal thickness in diastole was not affected by lercanidipine + losartan, while it was reduced by barnidipine + losartan (p < 0.01 vs baseline and p < 0.05 vs lercanidipine + losartan). Posterior wall thickness in diastole was decreased by both treatments, even if barnidipine + losartan were more effective in reducing it (p < 0.05 vs lercanidipine + losartan). Ratio of peak early diastolic filling velocity to peak filling velocity at atrial contraction was increased by barnidipine + losartan (p < 0.01 vs baseline and p < 0.05 in group to group comparison), but not by lercanidipine + losartan. Isovolumetric relaxation time was reduced by barnidipine + losartan (p < 0.01 vs baseline and p < 0.05 in group to group comparison), while lercanidipine + losartan did not affect it. LAVi was decreased by barnidipine + losartan (p < 0.05 vs baseline and p < 0.05 in group to group comparison), while lercanidipine + losartan did not affect it (Table 3).

### Adverse events

Ankle edema was complained by, or was clinically evident, in 3 patients treated with barnidipine and in 6 patients treated with lercanidipine. There was 1 reported case of rush with lercanidipine, 2 episodes of headache with barnidipine and 4 cases of headache with lercanidipine. No patients interrupted the study due to adverse events.

## Discussion

Barnidipine and lercanidipine are both third-generation CCBs indicated for the treatment of hypertension^20^. They are similar in reducing BP control, however, in literature, barnidipine has been reported to have a neutral tolerability profile with regard to insulin sensitivity^21^, indicating it might be particularly suitable for patients with metabolic syndrome, dyslipidemia, and impaired fasting glucose. This is confirmed by our data, in fact barnidipine did not worse glycemic control, and even improved LDL-C. Given that hypo-cholesterolemic agents were taken in both groups, and that their dosage was maintained stable during the study, the effects on lipid profile seem to be related to barnidipine itself.

Both barnidipine and lercanidipine improved acid uric, without differences between the two groups, this effect is probably due to the action of losartan, as already reported in literature in the COMFORT study where losartan decreased serum uric acid^22^.

Regarding the effects on echocardiographic parameters, comparative analysis of the effect of different dihydropyridines on left ventricle and coronary artery hypertensive changes have shown a better outcome for third generation compounds compared with first-generation or second-generation derivatives tested^23^. Among third generation dihydropyridines, barnidipine seems to have a positive effect on LV diastolic relaxation, as showed in a 12-week treatment period with barnidipine in addition to lifestyle modifications. No significant changes in LV structure, or systolic function were found^24^. Differently from this study, we recorded a positive effect of barnidipine on echocardiographic parameters, more effective than lercanidipine, with improvement of LV mass index, interventricular septal thickness in diastole, posterior wall thickness in diastole, isovolumetric relaxation time. This difference compared to the previous study is probably due to the longer duration of our study, 6 months compared to 3 months of the study by Angeli *et al*. The positive effect on echocardiographic parameters did not seem to be related to the decrease of BP, because both lercanidipine and barnidipine improved BP control, so we can suggest that barnidipine has a more pronounced end-organ protection probably related to the intrinsic characteristics of this compound.

To our knowledge, we are the first study to directly compare the effects on LVH of two different CCBs, and we think that the better effect of barnidipine in improving LVH should be considered in the clinical practice when choosing among different CCBs. Of course, our study has some limitations, as the short study period, longer study will be necessary to assess if the improvement of LVH can reduce the incidence of heart failure.

## Conclusions

Despite a similar improvement of BP control, barnidipine + losartan provided a greater improvement of echocardiographic parameters compared to lercanidipine + losartan.

## Statement of Human Rights

All procedures followed were in accordance with the ethical standards of the responsible committee on human experimentation and with the Helsinki Declaration of 1975, as revised in 2008. Informed consent was obtained from all patients for being included in the study. TRIAL REGISTRATION: *ClinicalTrials.gov* NCT02064218.

## Additional Information

**How to cite this article**: Derosa, G. *et al.* Barnidipine or Lercanidipine on Echocardiographic Parameters in Hypertensive, Type 2 Diabetics with Left Ventricular Hypertrophy: A Randomized Clinical Trial. *Sci. Rep.* **5**, 12603; doi: 10.1038/srep12603 (2015).

## Figures and Tables

**Table 1:** Main demographic, clinic and echocardiographic baseline characteristics of patients in the two treatment groups.

	Barnidipine + Losartan(n = 73)	Lercanidipine + Losartan(n = 71)
N	73	71
Age (years)	60.5 ± 8.9	60.7 ± 8.8
Sex (male/female)	36/37	36/35
BMI (Kg/m^2^)	28.5 ± 1.3	28.2 ± 1.1
SBP (mmHg)	150.2 ± 9.9	151.1 ± 10.3
DBP (mmHg)	96.0 ± 5.2	96.2 ± 5.4
HR (beats/min)	72.1 ± 8.0	74.5 ± 8.8
Fasting glucose (mg/dL)	122.4 ± 7.8	123.2 ± 7.9
HbA_1c_ (%)	6.8 ± 0.7	6.7 ± 0.6
Duration of diabetes (months)	8.8 ± 6.1	8.9 ± 6.2
Duration of hypertension (months)	3.5 ± 2.1	3.3 ± 2.0
Serum Creatinine (mg/dL)	0.98 ± 0.2	0.95 ± 0.1
eGFR (mL/min/1.73 m^2^)	78.4 ± 5.3	80.6 ± 5.4
Sodium (mEq/L)	142.6 ± 4.8	140.9 ± 4.0
Potassium (mEq/L)	4.1 ± 0.4	4.0 ± 0.3
TC (mg/dL)	208.1 ± 21.3	210.4 ± 22.9
Tg (mg/dL)	182.7 ± 59.1	184.5 ± 63.6
HDL-C (mg/dL)	44.1 ± 4.3	44.5 ± 4.1
LDL-C (mg/dL)	127.5 ± 18.5	129 ± 19.4
Acid Uric (mg/dL)	7.9 ± 1.0	7.8 ± 0.9
LVMI (g/m^2^)	134.1 ± 24.5	133.8 ± 24.2
IVSTd (mm)	10.98 ± 1.0	11.10 ± 1.1
PWTd (mm)	10.40 ± 1.0	10.47 ± 1.1
EF (%)	65.7 ± 4.6	66.1 ± 4.9
E/A ratio	0.88 ± 0.21	0.87 ± 0.20
IVRT (ms)	86.0 ± 13.5	86.8 ± 13.9
LAVi (mL/m^2^)	48.2 ± 17.1	48.7 ± 17.5

Data are means ± SD.

BMI: body mass index; SBP: systolic blood pressure; DBP: diastolic blood pressure; HR: heart rate; HbA_1c_: glycated hemoglobin; eGFR: estimated glomerular filtration rate; TC: total cholesterol; LDL-C: low density lipoprotein-cholesterol; HDL-C: high density lipoprotein-cholesterol; Tg: triglycerides; LVMI: left ventricular mass index; IVSTd: interventricular septal thickness in diastole; PWTd: posterior wall thickness in diastole; EF: ejection fraction; E/A ratio: ratio of peak early diastolic filling velocity to peak filling velocity at atrial contraction; IVRT: isovolumetric relaxation time; LAVi: left atrial volume index.

**Table 2:** Effects of the two treatments on metabolic parameters.

	Barnidipine + Losartan (n = 70)	Lercanidipine + Losartan (n = 69)
Sex (male/female)	35/35	35/34
Fasting glucose (mg/dL)	124.1 ± 8.2	123.8 ± 8.0
HbA_1c_ (%)	6.8 ± 0.7	6.7 ± 0.6
Serum Creatinine (mg/dL)	0.91 ± 0.1	0.92 ± 0.1
eGFR (mL/min/1.73 m^2^)	85.1 ± 6.7	84.3 ± 6.1
Sodium (mEq/L)	142.0 ± 4.6	141.3 ± 4.2
Potassium (mEq/L)	4.0 ± 0.3	4.1 ± 0.4
TC (mg/dL)	203.2 ± 20.1	211.3 ± 23.1
Tg (mg/dL)	166.3 ± 51.2	182.1 ± 62.5
HDL-C (mg/dL)	44.0 ± 4.0	44.3 ± 4.0
LDL-C (mg/dL)	115.1 ± 12.1*^	131 ± 22
Acid Uric (mg/dL)	7.4 ± 0.5*	7.5 ± 0.6*

Data are means ± SD.

*p < 0.05 vs baseline; ^p < 0.05 vs lercanidipine + losartan

HbA_1c_: glycated hemoglobin; eGFR: estimated glomerular filtration rate; TC: total cholesterol; LDL-C: low density lipoprotein-cholesterol; HDL-C: high density lipoprotein-cholesterol; Tg: triglycerides.

**Table 3:** Effects of two treatments on conventional echocardiographic parameters.

	Barnidipine + Losartan (n = 73)		Lercanidipine + Losartan (n = 69)	
LVMI (g/m^2^) Treatment	110.1 ± 16.1	*P *< 0.001 vs baseline *P *< 0.05 vs lerc/los	122.4 ± 19.5	*P*<0.05
IVSTd (mm) Treatment	9.05 ± 0.89	*P *< 0.01 vs baseline *P *< 0.05 vs lerc/los	10.5 ± 0.92	*Ns*
PWTd (mm) Treatment	8.54 ± 0.8	*P *< 0.01 vs baseline*P *< 0.05 vs lerc/los	9.58 ± 0.9	*P *< 0.05
EF (%) Treatment	66.3 ± 4.7	*Ns*	66.4 ± 5.6	*Ns*
E/A ratio Treatment	1.01 ± 0.26	*P *< 0.01 vs baseline *P *< 0.05 vs lerc/los	0.92 ± 0.21	*Ns*
IVRT (ms) Treatment	77.2 ± 11.9	*P *< 0.01 vs baseline *P *< 0.05 vs lerc/los	83.2 ± 12.5	*Ns*
LAVi (mL/m^2^)	37.2 ± 11.4	*P *< 0.05 vs baseline *P *< 0.05 vs lerc/los	43.5 ± 13.1	*Ns*

Data are means ± SD.

LVMI: left ventricular mass index; IVSTd: interventricular septal thickness in diastole; PWTd: posterior wall thickness in diastole; EF: ejection fraction; E/A: ratio of peak early diastolic filling velocity to peak filling velocity at atrial contraction; IVRT: isovolumetric relaxation time; LAVi: left atrial volume index; lerc/los: lercanidipine + losartan.
